# Safety of Allogeneic Canine Adipose Tissue-Derived Mesenchymal Stem Cell Intraspinal Transplantation in Dogs with Chronic Spinal Cord Injury

**DOI:** 10.1155/2017/3053759

**Published:** 2017-05-15

**Authors:** Cláudia Cardoso Maciel Escalhão, Isalira Peroba Ramos, Camila Hochman-Mendez, Tais Hanae Kasai Brunswick, Sergio Augusto Lopes Souza, Bianca Gutfilen, Regina Coeli dos Santos Goldenberg, Tatiana Coelho-Sampaio

**Affiliations:** ^1^Institute of Biomedical Sciences, Federal University of Rio de Janeiro, Ave. Carlos Chagas Filho 373, Sala B1-011, Cidade Universitária, Ilha do Fundão, 21941-590 Rio de Janeiro, RJ, Brazil; ^2^Institute of Biophysics Carlos Chagas Filho, Federal University of Rio de Janeiro, Ave. Carlos Chagas Filho 373, Sala G2-053, Cidade Universitária, Ilha do Fundão, 21941-590 Rio de Janeiro, RJ, Brazil; ^3^Department of Radiology, University Hospital Clementino Fraga Filho and Federal University of Rio de Janeiro, Rua Prof Rodolpho Paulo Rocco, n 255, Sala ss29, Cidade Universitária, Ilha do Fundão, 21941-913 Rio de Janeiro, RJ, Brazil; ^4^National Center Structural Biology and Bio-imaging, Federal University of Rio de Janeiro, Ave. Carlos Chagas Filho 373, Sala M1-22, Cidade Universitária, Ilha do Fundão, 21941-590 Rio de Janeiro, RJ, Brazil

## Abstract

This is a pilot clinical study primarily designed to assess the feasibility and safety of X-ray-guided percutaneous intraspinal injection of allogeneic canine adipose tissue-derived mesenchymal stem cells in dogs with chronic spinal cord injury. Six dogs with chronic paraplegia (≥six months) were intraparenchymally injected with allogeneic cells in the site of lesion. Cells were obtained from subcutaneous adipose tissue of a healthy dog, cultured to passage 3, labeled with ^99m^Technetium, and transplanted into the lesion by percutaneous X-ray-guided injection. Digital X-ray efficiently guided cell injection as ^99m^Technetium-labeled cells remained in the injection site for at least 24 hours after transplantation. No adverse effects or complications (infection, neuropathic pain, or worsening of neurological function) were observed during the 16-week follow-up period after transplantation. Three animals improved locomotion as assessed by the Olby scale. One animal walked without support, but no changes in deep pain perception were observed. We conclude that X-ray-guided percutaneous intraspinal transplantation of allogeneic cells in dogs with chronic spinal cord injury is feasible and safe. The efficacy of the treatment will be assessed in a new study involving a larger number of animals.

## 1. Introduction

Severe spinal cord injury (SCI) that interrupts supraspinal input to thoracolumbar spinal circuits may lead to permanent disability in humans and dogs. Treatment of acute SCI focuses on surgical decompression and stabilization of the body/spinal cord instability and on minimizing the progression of the secondary damage to favor eventual endogenous repairing. If the disability persists, it progresses to the chronic phase, when the loss of deep pain perception is an important negative prognosis for the return of function [1]. In this condition, there is no standard treatment to recover locomotion. In the chronic phase, experimental treatments aim mainly at promoting synapse plasticity, axonal sprouting, or regeneration. In addition, networks of neurons in the spinal cord may retain an intrinsic ability to oscillate and generate coordinated and rhythmic motor output [2, 3]. These circuits, called central pattern generators (CPG), could alternatively be stimulated to promote effective motor function [3, 4].

The use of mesenchymal stem cells (MSC) in regenerative medicine has provided new strategies for tissue repair and a potential for novel treatments [5]. Mesenchymal stem cells have been successfully isolated from canine umbilical cord blood [6, 7], Wharton's jelly [8], bone marrow [9], and adipose tissue [10, 11]. MSCs harvested from these sources are selected through their adherence to plastic and self-renewal capacity in culture systems under specific conditions [12]. Adipose tissue is the most abundant and readily available source of MSCs [13], where they reside in a perivascular niche, coexisting with pericytes and endothelial cells [14]. Adipose tissue-derived MSCs (AT-MSCs) produce neurotrophic factors and may protect against hypoxia-ischemia and prevent glutamate neurotoxicity [15, 16]. In rodent models of acute spinal cord injury, local transplantation of AT-MSCs promoted nervous tissue protection and functional recovery [17, 18].

In dogs, AT-MSCs, previously induced to neural cells or not, have been used to treat experimental spinal cord injury during the acute phase [8, 19, 20]. All these studies reported that cell transplantation promoted neurological gains and improved locomotion. In addition, morphological analyses revealed reductions in lesion size, astrogliosis, and inflammatory reactions, as well as a fostering of neuronal survival and tissue preservation. Despite the promising effects of treating acute spinal cord injury in dogs with AT-MSCs, little has been done in chronic lesions. Only very recently, Lee and coworkers [21] reported benefits of AT-MSCs in combination with chondroitinase ABC in a model of balloon compression lesion in beagles. Until now, there are no studies with these cells in dogs bearing chronic natural lesions to support future clinical validation.

In the present study, we assessed the feasibility and safety of allogeneic canine AT-MSC transplantation in dogs bearing natural spinal cord lesions provoked by trauma or intervertebral disc disease and not presenting deep pain perception for at least six months. In addition, our study validated percutaneous cell delivery guided by digital X-ray imaging as a valuable method of nonsurgical cell transplantation in the clinical setting.

## 2. Methods

### 2.1. Ethics Statement

Dogs were maintained according to the recommendations of the Guide for the Care and Use of Laboratory Animals (NIH) and the Brazilian College of Animal Experimentation (COBEA). The Ethics Committee for Animal Use of the Federal University of Rio de Janeiro (Rio de Janeiro, Brazil) approved all experiments (01200.001568/2013-87). All owners provided written consent for the participation of their dogs in this study, which includes authorization for publishing data obtained in the study.

### 2.2. Animals, Inclusion Criteria, and Study Design

**Figure 1:**
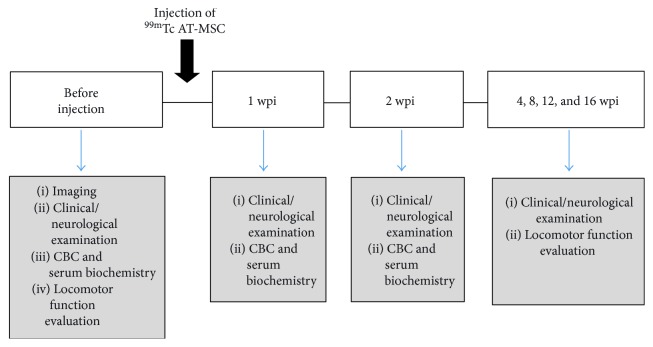
Experimental design. Chronogram of the experimental procedures carried out before and after injection of ^99m^TC AT-MSCs. CBC, complete blood count; wpi, weeks postinjection.

Six dogs (four males and two females; mean age 7.17 years old; range 6–12 yrs.) were enrolled in the study (Table 1). The inclusion criteria were defined as severe chronic SCI, from the thoracic to the lumbar level, caused by vertebral fracture, luxation, or intervertebral disc disease (IVDD) paraplegia without cranial response to noxious stimuli applied to the interdigital space in the pelvic limb with a hemostatic device. Out of the six dogs included, three had had surgery before the injection of AT-MSCs (animals 2, 5, and 6) and three had not (animals 1, 3, and 4). A healthy dog was used for cell isolation. The experimental design of the study is summarized in Figure 1.

### 2.3. AT-MSC Isolation, Culture, and Expansion

Adipose tissue was aseptically collected under anesthesia from subcutaneous fat of a two-year-old dog during castration, with permission from the owners. Tissue was minced into small pieces for enzymatic digestion with 0.2% collagenase type I (Worthington Biochemical Corporation, Lakewood, NJ, USA) for 50 min at 37 °C. Cells were plated and expanded in Dulbecco's Modified Eagle Medium (DMEM) low glucose supplemented with 20% fetal bovine serum (FBS, Thermo Fisher Scientific, Waltham, MA, USA), 2 mM L-glutamine, and 1% penicillin-streptomycin. Cultures were expanded, and experiments were performed at passage 3.

### 2.4. Immunophenotypic Profile of Canine AT-MSCs

Expression of cell surface molecules of canine AT-MSCs at passage 3 was evaluated by flow cytometry. Briefly, cells were harvested from cell flasks by enzymatic digestion with 0.25% trypsin-EDTA (Sigma-Aldrich). Next, cells were stained with conjugated antibodies (anti-CD45-Alexa 647, anti-CD19-FITC, anti-CD14-PE, anti-CD73-APC, anti-CD90-APC, anti-CD105-PE, and anti-CD44-FITC; all from eBioscience, San Diego, CA, USA) diluted in PBS containing 0.5% BSA. AT-MSCs were incubated for 20 min in the dark at 4°C. Following incubation, cells were washed with PBS and centrifuged at 300 ×g for 5 min. Cell pellets were resuspended with PBS for data acquisition using a BD FACSAria™ II flow cytometer. Data were analyzed using FlowJo software (FlowJo LCC, Ashland, OR, USA).

### 2.5. Cell Differentiation

For adipogenic differentiation, cells were submitted to a 21-day culture protocol with DMEM supplemented with 10% FBS, penicillin-streptomycin, 10 *μ*M dexamethasone, 200 *μ*M indomethacin, 10 *μ*g/mL human insulin, and 0.5 mM isobutyl-methylxanthine (all reagents from Sigma-Aldrich). After this period, cells were fixed and stained with oil red O.

Osteogenic differentiation was induced in DMEM supplemented with 10% FBS, penicillin-streptomycin, 0.01 *μ*M dexamethasone, 10 mM *β*-glycerophosphate, and 0.5 *μ*M ascorbic acid. After this period, cells were fixed and used for the determination of alkaline phosphatase (ALP) activity and von Kossa staining for the detection of mineralization.

Alkaline phosphatase activity was measured using p-nitrophenylphosphate (Sigma-Aldrich) as a substrate in alkaline buffer solution (20 mM p-nitrophenylphosphate + 100 mM diethanolamine 98% + 10 mM MgCl_2_, pH 9.5). Samples were removed from culture, washed in PBS, permeabilized with 0.5% aqueous Triton X-100 (Sigma-Aldrich), and incubated with substrate solution for 30 min at 37°C. The same volume of EDTA solution (0.1 M EDTA in 1 M NaOH) was added. Optical density readings were taken using an ELx 800 absorbance reader (BioTek Instruments Inc., Winooski, VT, USA) at 405 nm.

For von Kossa staining, monolayers were incubated with 2% silver nitrate solution in distilled water for 1 h, protected from light, washed five times with distilled water, and exposed to UV light for 10 min. To quantify the degree of mineralization, monolayers were photographed using an inverted microscope (Nikon Eclipse TS100, Nikon, Tokyo, Japan) equipped with an EC3 digital camera (Leica, Wetzlar, Germany). Mineralization foci were quantified in 15 random fields using ImageJ software (U.S. National Institutes of Health, Bethesda, MD, USA) and expressed as a percentage of the total area.

Chondrogenic differentiation was induced in DMEM supplemented with 1% FBS, penicillin-streptomycin, 10 ng/mL TGF-*β*3, 6.25 *μ*g/mL insulin, 6.25 *μ*g/mL transferrin, and antibiotics. Cells were suspended at 10^7^ cells/mL, and 10 *μ*L droplets were carefully placed on a plate and maintained at 37°C for 2 h, followed by an addition of 500 *μ*L of chondrogenic medium. The medium was changed every three days, and the micromasses formed were collected at day 21 of culture. Micromasses were cryopreserved in increasing sucrose concentrations, embedded in OCT compound (Tissue-Tek®, Sakura Finetek, Alphen aan den Rijn, The Netherlands), and stored at −80°C. Four-micrometer-thick sections were cut using a cryostat (Leica CM 1800s, Germany) and stained with alcian blue and neutral red.

### 2.6. Cell Labeling with ^99m^Tc

Approximately, 10^7^ cells were labeled with ^99m^Tc. Briefly, 500 *μ*L of sterile SnCl_2_ solution was added to the cell suspension in 0.9% NaCl, and the mixture was incubated for 10 min at room temperature. Next, 10 mCi ^99m^Tc was added and the incubation was continued for 10 min. After centrifugation (500 ×g for 5 min), the supernatant was removed, cells were washed in saline solution, and the pellet was resuspended in saline solution. The preparation and labeling of cells were all performed in a laminar flow.

### 2.7. Clinical Procedure

Premedication was done with intravenous chlorpromazine (1 mg·kg^−1^). Anesthesia was induced with propofol (5 mg·kg^−1^ IV) and maintained with isoflurane in oxygen. ECG and CO_2_ partial pressure were monitored.

**Figure 2:**
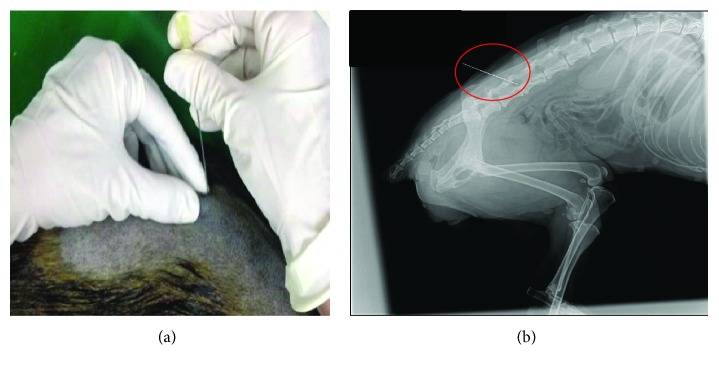
Localization of the injection site. (a) Photograph depicting the procedure of percutaneous injection. (b) X-ray image of the vertebral column, highlighting the affected intervertebral space.

To visualize the intervertebral space for needle placement, dogs underwent an X-ray exam (Figure 2). Following lesion localization, ^99m^Tc-labeled AT-MSCs were intralesionally injected in each dog. Scintigraphy images were taken 1 and 24 h after injection to assess localization of AT-MSCs.

### 2.8. Postoperative Procedures

Bladder management and nursing care were done to prevent urinary infection and decubitus ulcers. The rehabilitation procedures included massage, passive exercises (flexor reflex stimulation and bicycle movements), and active exercise (sit-to-stand with sling) 10–15 min per day across the 16-week follow-up period.

### 2.9. Imaging

Scintigraphy scans were performed using a GE Millennium® camera system (General Electric Medical Systems, Milwaukee, WI, USA). Images were acquired 1 and 24 h after cell transplantation, with the animal in the supine position. Whole body images were acquired for 5 min in anterior and posterior views using a dual-head whole body scanner with a high-resolution, low-energy collimator. Planar images of the spine were acquired for 10 min using a 256 × 256 matrix in anterior and posterior views. A germanium flood source was used to draw a shadow of the body of the animal to better visualize ^99m^Tc AT-MSC uptake. Single-photon emission computed tomography (SPECT) was also performed 1 h after cell transplantation. Image reconstruction was done using a GE Xeleris™ processing workstation.

### 2.10. Blood Tests

Complete blood count (CBC) and serum chemistry tests were performed. Alanine aminotransferase (ALT), aspartate aminotransferase (AST), and alkaline phosphatase (ALP) levels were measured to determine liver damage. Serum levels of creatinine and urea were determined to evaluate renal function. Blood was taken before and at one and two weeks after AT-MSC injection. Blood tests were performed in private certified laboratories chosen according to the owner's convenience.

### 2.11. Clinical and Neurological Exams

Animals were submitted to clinical evaluation at least once before cell injection, immediately before injection and 1, 2, 4, 8, 12, and 16 weeks after surgery. In addition to clinical examination, dogs received specific neurological evaluation, which included segmental reflexes, postural reactions, sensitive analysis, and an additional exam of cranial nerves.

### 2.12. Functional Evaluation

Locomotor function was evaluated using the Olby scale (0–14: 0 indicates complete paraplegia and 14 indicates normal gait). Dogs were filmed walking on a treadmill or on an antislip floor in a position where the hind limbs could be clearly viewed. Animals were monitored before and four, eight, 12, and 16 weeks after AT-MSC injection.

### 2.13. Statistical Analysis

Data are presented as mean ± SEM. Data were analyzed using one-way analysis of variance (ANOVA) and post hoc analysis was performed using the Bonferroni multiple comparison test or the Student *t*-test. Analyses were performed using GraphPad Prism 6.0 software (GraphPad Software Inc., San Diego, CA, USA). Data were considered significant at *p* < 0.05.

## 3. Results

### 3.1. Morphological and Immunophenotypic Characterization of Canine AT-MSCs

**Figure 3:**
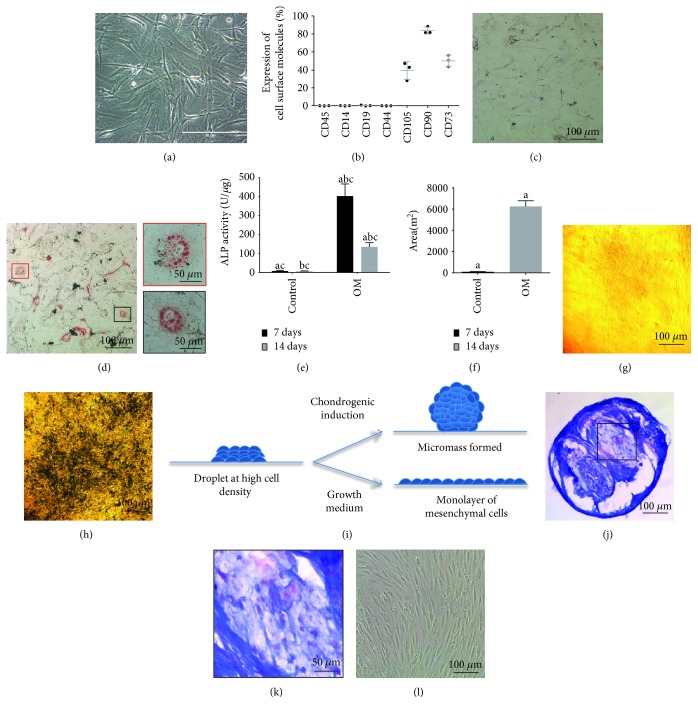
Characterization of canine AT-MSCs. (a) Phase-contrast micrograph showing AT-MSCs adhered to a plastic culture flask at passage 3. (b) Immunophenotypic profile. Flow cytometry analyses were carried out for each marker separately. The percentages of positive cells are shown in the graph. (c, d) Adipogenic differentiation. Cells were grown in regular (c) or adipogenic (d) medium for 21 days, fixed, and stained with oil red O to evaluate the presence of red-colored lipid drops in the cytoplasm. The insets correspond to higher magnifications of areas boxed in (d). (e–h) Osteogenic differentiation. Alkaline phosphatase (ALP) activity was measured after 7 or 14 days of culture in osteogenic medium (right bars) or regular medium (left bars), as indicated in (e). Bars labeled with the same letters are significantly different (*p* ≤ 0.05). Von Kossa staining revealed more mineralization foci at day 7 in induced cells (f). (g, h) show the absence or presence of mineralization deposits in osteogenic and regular medium, respectively. (i–l) Chondrogenic differentiation. (i) Scheme representing the micromass assay. (j) Micromass generated after 21 days in chondrogenic medium after being processed, cut, and stained with alcian blue and counterstained with neutral red to evidence cell nuclei. (k) Selected area in (j), showing the typical structure of cartilage tissue with nuclei eccentrically placed in lacunae and an alcian blue-stained matrix revealing the presence of glycosaminoglycans. (l) No micromasses were observed after 21 days in control medium. Conversely, the droplets were disassembled giving rise to a homogeneous cell monolayer.

Canine cells isolated in this study were characterized as mesenchymal cells both morphologically and immunophenotypically. While attached to plastic at passage 3, they presented a thin and elongated fibroblastoid phenotype, typical of mesenchymal cells (Figure 3(a)).

Immunophenotypic analyses of canine AT-MSCs by flow cytometry showed a large proportion of cells expressing CD105 (39.31%), CD90 (83.83%), and CD73 (50.7%) molecules. Additionally, no contamination by cells of hematopoietic origin, expressing CD45, CD14, CD19, and CD44, was observed.  Figure 3(b) shows the percentage of cells labeled each with antibody.

### 3.2. Differentiation Potential of Canine AT-MSCs

In order to confirm that AT-MSCs isolated in this work exhibited the potential to differentiate into multiple mesenchymal lineages, we tested their adipogenic, osteogenic, and chondrogenic potentials. Cells grown in adipogenic medium acquire a round shape and accumulate oil red O-positive lipid drops in the cytoplasm (Figure 3(c)). On the other hand, cells cultivated in control medium for the same period did not exhibit positivity for oil red O (Figure 3(d)).

The osteogenic potential of canine AT-MSCs was investigated by assessing alkaline phosphatase activity and von Kossa staining. After 7 days in osteogenic medium, cells presented alkaline phosphatase activity while cells in control medium did not (Figure 3(e)). At day 14, ALP activity decreased 60% in comparison with the activity at day 7, which is compatible with the peak of osteogenic differentiation being at the seventh day after induction. Osteogenesis was additionally confirmed by investigating mineralization foci in the cultures at day 7. Von Kossa staining was carried out as described in Section 2. A representative picture as well as a quantification of the mineralization areas in 15 fields is shown in Figures 3(f)–3(h).

Chondrogenic differentiation was assessed using the micromass assay, where highly concentrated droplets of cells are seeded onto culture dishes and cultivated in the presence or in the absence of chondrogenic medium. After 21 days in culture, only cells in differentiation medium formed spherical micromasses. The micromasses were manually collected and processed to reveal the formation of a cartilage-like tissue, containing alcian blue-positive glycosaminoglycans (Figures (i)–3(l)). This result confirmed the chondrogenic potential of canine AT-MSCs.

### 3.3. Analysis of Cell Distribution by Scintigraphy

**Figure 4:**
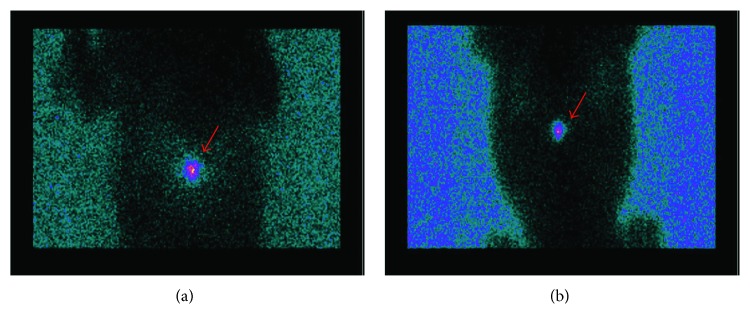
Scintigraphy of dogs injected with ^99m^Tc-labeled adipose tissue-derived mesenchymal stem cells. Representative scintigraphy images are shown for animal 4 and were obtained either 1 (a) or 24 hours (b) after cell injection. The arrows point to single radioactive spots (color). Body contours were revealed by using a germanium flood source. Cells are located in the thoracic spine at both 1 and 24 h after transplantation. Transplanted cells remained at the injection site for the first 24 h after transplantation.

The biodistribution of AT-MSCs was assessed by scintigraphy. In the present study, we used digital X-ray-guided percutaneous injection of the cells, which was not a validated method for cellular delivery into the spinal cord. For this reason, it was important to confirm that the graft reached the aimed location within the spine and that it remained there for at least 24 hours, When the whole body was scanned one hour after injection, 5 out of 6 animals presented only one single-labeled spot, whose location was compatible with the desired spinal segment.  Figure 4(a) shows the scintigraphy of animal 4 one hour after cell transplantation at the thoracic spine (T12-T13). One (animal 1) out of 6 animals presented one major spot at the injection site but also displayed scattered labeling throughout the body, with focuses in regions compatible with the lungs, liver, and kidney. Such secondary spots represented 5–10% of the total radioactivity. Signal diffusion was probably due to the accidental hit of a small blood vessel. Animals were submitted to a second scintigraphy after 24 h, and the spots observed were similar to those obtained after 1 hour for each animal. These results show that after transplantation, cells did not diffuse to other locations within the body (Figure 4(b)).

### 3.4. Safety of Intraparenchymal AT-MSC Injection

In order to evaluate the safety of injecting AT-MSCs directly into the spinal cord, we have performed two assessments, namely, a detailed clinical/neurological examination and a set of blood tests. No cell-related adverse events (e.g., infection and neuropathic pain) were observed during the procedure or follow-up period, supporting the notion that cell transplantation is safe. On the other hand, none of the dogs recovered deep pain perception or bladder function, although dog 3 recovered ambulation (see Section 3.5). Noteworthy were the observations that animal 2 presented a slight recovery of proprioception, which shifted from absent before transplantation to diminished after 4 weeks. In addition, this same dog stopped self-mutilating the posterior digits, suggesting a reduction of paresthesia.

**Figure 5:**
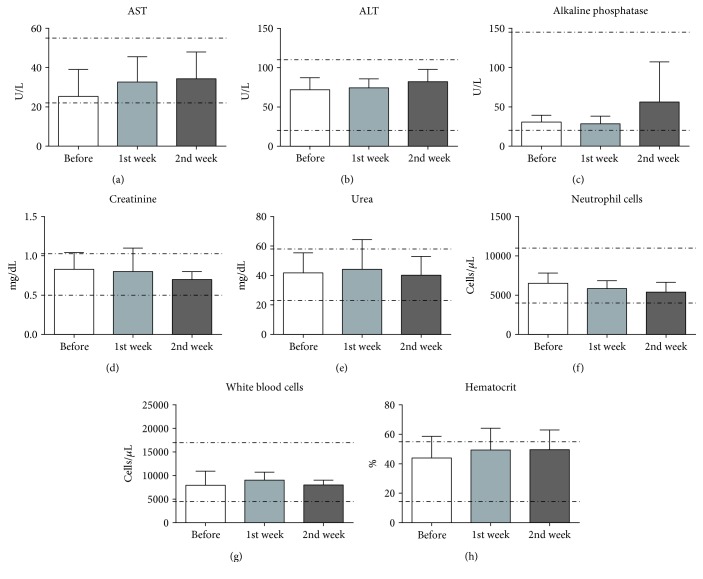
Evaluation of biochemical and hematological markers. AST (a), ALT (b), ALP (c) (U/L), creatinine (d), and urea (e) (mg/dL) levels before and one and two weeks after AT-MSC transplantation. Complete blood count (CBC) of animals treated with AT-MSCs: neutrophils (f), total white blood cells (g), and hematocrit (h). Dotted lines show the normal range of values (reference values). No significant differences were found in serum and blood parameters across time (Student's *t*-test, *p* = ns).

Blood tests were carried out before and 1 and 2 weeks after injection. None of the biochemical markers of liver and kidney functions were affected by the transplantation procedure. Values for AST, ALT, alkaline phosphatase, creatinine, and urea remained within the normal range (Figures 5(a)–5(e)). The analysis of cell blood counts did not show alterations (Figures 5(f)–5(h)), confirming lack of evidence for deleterious effects of intraspinal injection of AT-MSCs in dogs.

### 3.5. Functional Evaluation of Locomotor Activity

**Figure 6:**
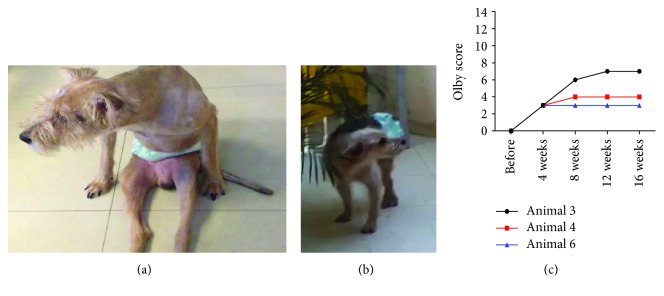
Locomotor function evaluation. Animals were evaluated before and after AT-MSC transplantation by the Olby scale. Animal 3 before (a) and after (b) AT-MSC transplantation. (c) Olby scores obtained for the three dogs that showed functional improvements (animals 3, 4, and 6).

The locomotor function was evaluated using the Olby scale, which is a practical test to detect eventual functional gains of the treatment but which has the limitation of not assessing possible differences between the affected hind limbs. Animal 1, who had IVDD between L5 and L6 one year before transplantation, received an Olby score of 4 before treatment and did not present improvement at the end of the study. Animal 2, IVDD between T13 and L1 one year before transplantation, received scores of 3 before and after transplantation, despite the clinical observation of improvements in postural reaction (proprioception) and interruption of self-mutilating after AT-MSC transplantation. Dog 3, who had a fracture luxation at T13-L1 without surgical stabilization or decompression and was paraplegic for six months before treatment, showed a progressive increase in locomotor function. The Olby score increased from 0 before treatment to 7, 16 weeks after AT-MSC transplantation. Animal 4, IVDD between T12 and T13 six months before transplantation, also improved locomotor function, changing the Olby score from 0 before treatment to 4 after AT-MSC transplantation. Dog 5, IVDD between T11 and T12, submitted to decompression surgery two years before without report of functional improvement, received Olby scores of 3 before and after cell treatment. Finally, dog 6, who had a T12 fracture followed by surgery with decompression and stabilization one year before transplantation, received Olby scores of 0 before transplantation and 3 after, 4 weeks after cell implant. The score of 3 remained unchanged between 4 and 16 weeks. Out of the 6 dogs included in the study, none presented reduction in the Olby score and three presented improvements (Figure 6). Only one (animal 3) of the dogs in the study regained the ability to walk.

## 4. Discussion

In this work, we set out to investigate the effects of an intraspinal injection of allogeneic canine AT-MSCs in dogs with natural chronic spinal cord injury. The study was designed as a pilot clinical test with two primary goals, to ascertain the feasibility and the safety of the procedure and the secondary goal of verifying a possible beneficial effect of the treatment. Our study is the first to use adult stem cells in dogs bearing natural lesions that had progressed to the chronic phase. This is important because chronically injured animals displaying stable functional deficits are thought to be the major population to benefit of a future cell therapy-based standard treatment for spinal cord injury in the veterinary set.

The feasibility of the procedure needed to be tested since we chose a novel and rather unrefined procedure for cell delivery, namely, a percutaneous injection guided by a portable digital X-ray device. Previous works had used fluoroscopic-guided percutaneous injection [22] or open surgery for cell grafting [23]. By scintigraphy imaging, we found that cells previously labeled with ^99m^Tc were delivered into the desired region within the lesioned segment of the spinal cord. The observation that cells remained within the injection area after 1 and 24 hours supported the notion that they reached the solid tissue of the spinal parenchyma instead of the cerebrospinal fluid, from which they would have spread along the axis of the cord. Our procedure reduces the complexity of intraspinal cell transplantation, since it can be performed in a simple way with a percutaneous injection, basically only requiring the equipment necessary for deep anesthesia.

Previous works have studied the effects of canine AT-MSCs in dogs with spinal cord injury [19–21]. While their overall conclusion was that the treatment promoted functional recovery and tissue preservation, little focus was given to possible detrimental clinical effects of allogeneic cell transplantation into the experimental subjects. Since the absence of detriments is a fundamental point if one wants to move forward to clinical approval of cell therapy, here, we included a detailed evaluation of the clinical status of the experimental animals. Analyses of serum markers of liver and kidney functions showed no alterations at one or two weeks after transplantation, while the values obtained remained within the range of the standard values for each parameter. A similar behavior was obtained when cell blood counts were analyzed. In addition, a detailed neurological examination reported no functional deterioration in any of the animals, which indicates that the intraspinal injection of AT-MSCs was harmless for dogs. We opted for presenting blood analyses results as the means obtained for the whole group of animals because such average represented each dog individually. In none of the six animals evaluated, we found values for CBC or biochemical markers falling out of the standard ranges determined for each parameter.

Mesenchymal stem cells are known to negatively modulate the immune system [24, 25], which is the reason for them to be suitable for allogeneic transplantation. Such immunosuppressive effects may account for the neuroprotection they confer when injected within the first days after SCI [18, 26]. The immune reaction following SCI in dogs has been characterized during the acute and subacute phases as a predominantly innate response, mainly mediated by resident microglia and not involving consistent infiltration of peripheral T cells or B lymphocytes [27, 28]. This is distinct from the occurring in rodents, where the adaptative immune response plays a major role [29, 30]. Moreover, in rodents, peripheral infiltration of inflammatory cells after SCI extends up to the chronic phase, peaking at two months after injury [31]. A similar phenomenon in dogs has not been searched for. However, the fact that, contrary to rodents, dogs do not develop a notable adaptative response, suggests that the predictable immunosuppressive effect of AT-MSCs should not play a role in the context of a chronic lesion as in the present study. Although we have not carried out a direct assessment of inflammation, the lack of alteration in leukocyte counts is in accordance with this interpretation. Interestingly, Lee and coworkers, who injected AT-MSCs in dogs with experimentally inflicted SCI in the chronic phase, reported even a proinflammatory effect of AT-MSCs detected as enhancements in levels of COX2 and TNF-*α* [21].

The third aim envisaged here was to test possible functional benefits of transplanted AT-MSCs. Despite the drawbacks of the reduced number of animals (*n* = 6) and the heterogeneity of the lesions, the fact that all dogs included in the study had stabilized locomotion deficits for at least six months prior to injection allowed for the detection of eventual motor gains after treatment. The Olby scale was used to monitor the locomotor performance. The Olby scale, which is based on the Basso, Beattie, and Bresnahan (BBB) locomotor rating scale [32], had been originally designed in response to the lack of a sufficiently sensitive method for detecting small changes in locomotor ability of dogs that could be used in clinical studies [33], although it does not assess possible differences between the affected hind limbs. It has the advantage of providing a way to quantify the progress in ambulation, which, in a clinical setting, is the most relevant parameter. Recently, the same research group [34] developed a method that quantifies stepping ability and coordination in dogs with a wide range of severities of thoracolumbar spinal cord injury. Thus, the scale has also been validated for monitoring functional progress in other types of injuries, including spinal cord injury, as used here.

Out of the six dogs included in our study, two (animals 1 and 5) did not present any sign of functional improvement after treatment. Animal 1 had a lumbar injury at L5-L6. The fact that only the tip-end of the spinal cord is present at this segment might explain the lack of beneficial effects in animal 1, who probably lost ambulation due to some additional injury. Animal 5 was by far the older, heavier, and most chronically injured dog included. Animal 2 did not show progress in the Olby score but presented a slight improvement in postural reaction, which moved from absent to minimal. More importantly, dog 2 stopped intense self-mutilation after treatment, which is an indication of recovery in sensitivity. The other three dogs, 3, 4, and 6, showed progress in the evaluation by the Olby scale. Animals 4 and 6 moved from 0 to 4 and 3, respectively. Although the final scores reached were not sufficient to permit independent ambulation, these dogs recovered variable degrees of joint movements, which were absent before cell injection. Finally, animal 3 progressed from 0 to 7 and regained independent gait. In the three cases of functional progress, the locomotor gains were obtained within 8 weeks after transplantation and tended to stabilize between 8 and 16 weeks, opening the possibility that a second injection could contribute to further recovery. Curiously, the three animals that improved the Olby scores were those starting with score 0. One explanation for this is that the recovery promoted by AT-MSCs might be restricted to the recuperation of a small number of fibers, which will only result in the control of articulations moved by muscles simultaneously innervated by various groups of fibers. Another interesting observation was that the two animals bearing fractures showed progress in the Olby scale, while out of the four dogs bearing IVDD, only one showed progress.

Recovery of motor function after SCI can be attributed to several mechanisms. In most experimental studies, using models of acute spinal cord injury, benefits promoted by AT-MSCs are thought to result from the secretion of molecules that contribute to protect the nervous tissue to mitigate secondary damage, such as neurotrophic factors, anti-inflammatory cytokines, and extracellular matrix molecules [18, 26]. In chronic injury, however, it is unlikely that cells will promote neuroprotection. Instead, AT-MSCs are urged to play a proregenerative function, either mediated by replacing lost cells, particularly oligodendrocytes, which would facilitate remyelination of uninjured axons, or mediated by some degree of neural plasticity, involving axonal sprouting or the formation of new circuits. In the present work, we have not accessed the spinal tissue because animals were clinical subjects and remained alive after completion of the study. Therefore, it was not possible to ascribe the recovery to any of these specific regenerative mechanisms, although the lack of recovery in deep pain perception in all animals suggests that recovery preferentially attained local motor circuits. Nevertheless, independently of whether recovery occurred due to the stimulation of local generated circuits or to axonal regeneration, such recovery happened only after the therapy was delivered to chronically paralyzed dogs. Therefore, we can establish a correlation between the injection of AT-MSCs and the functional progress. We propose that further studies using AT-MSCs will contribute for the development of an efficient new therapy to treat spinal cord injury in dogs.

## Figures and Tables

**Table 1:** Summary of clinical data of dogs with SCI transplanted with allogeneic adipose tissue-derived mesenchymal stem cells.

Animal	Breed/sex	Age (yrs)	Weight (kg)	History	Lesion site	Exam	Time postlesion (yrs)
1	Beagle/M	6	12	IVDD	L5-L6	CT	1
2	Daschund/F	7	10	IVDD	T13-L1	CT	1
3	Mongrel/M	6	13	Trauma	T13-L1	XR	0.5
4	Whippet/F	6	11	IVDD	T12-T13	CT	0.5
5	Pit bull/M	12	35	IVDD	T11-T12	CT	2
6	Poodle/M	6	10	Trauma	T12	CT	1

M: male; F: female; IVDD: intervertebral disc disease; CT: computed tomography; XR: X-ray.
